# Extemporaneous Compounding and Physiological Modeling of Amlodipine/Valsartan Suspension

**DOI:** 10.1155/2021/6695744

**Published:** 2021-03-12

**Authors:** Wafa' J. Aabed, Asma H. Radwan, Abdel Naser Zaid, Naser Y. Shraim

**Affiliations:** Department of Pharmacy, College of Medicine and Health Sciences, An-Najah National University, Nablus, State of Palestine

## Abstract

**Method:**

Amlodipine/valsartan extemporaneous suspension was prepared from available commercial tablets such as Valzadepine^®^. The dissolution profiles for the extemporaneous preparation and the commercial tablet were determined in different pH media. The physical, chemical, and microbial stability of the compounded formulation was evaluated over one-month period at room temperature. Moreover, *in silico* modeling using GastroPlus™ software was used to build absorption models for both drugs based on the *in vitro* dissolution data. The simulated plasma profiles for both active ingredients were compared with the *in vivo* plasma profiles to examine the similarity of the extemporaneous suspension and the commercial tablets.

**Results:**

The amlodipine/valsartan extemporaneous suspension was successfully prepared with acceptable organoleptic properties. The suspension was stable for four-week period preserving its physical and chemical features. The release profiles of valsartan and amlodipine from the suspension were similar to those from source tablet Valzadepine^®^. *In silico* modeling predicted the similarity of the extemporaneous suspension and the commercial tablets.

**Conclusion:**

Amlodipine/valsartan extemporaneous suspension could be prepared from available commercial tablets. Moreover, GastroPlus™ can be applied along with the *in vitro* dissolution in order to affirm similarity in extemporaneous compounding situations.

## 1. Introduction

Among all formulations, oral preparations are still of much of interest. When considering pediatrics and geriatrics with swallowing difficulties, liquid preparations are the most preferred formulations. In case of absent liquid preparation of an active ingredient, health care providers tend to split or crush the oral solid dosage form ignoring its safety and efficacy [1]. Weight uniformity of the subdivided tablets cannot be guaranteed all the time even for scored tablets; this may lead to serious complications especially in case of narrow therapeutic window drugs [2]. Moreover, crushed tablets that are used for preparing an extemporaneous suspension do not follow any stability or bioavailability testing [34], which may lead to further confusion whether these crushed tablets preserve their efficacy or safety issues which may lead to serious complications.

Nowadays, there is an increasing interest in developing new formulations of marketed agents to keep up with the market need. Since there is a lack in availabilty of liquid preparations of antihypertensive medications products, there is a need to develop and compound such preperations.

Amlodipine is a calcium channel blocker that causes the arteries to dilate leading to a reduction in blood pressure (BP). Besides, valsartan is an angiotensin receptor blocker (ARB) that prevents angiotensin, which is known for its ability to constrict blood vessels, from binding to angiotensin receptors, therefore blocking these receptors leading to a decrease in blood pressure [4]. Combination therapy of amlodipine and valsartan was significantly more effective in lowering BP than using amlodipine or valsartan alone [5, 6]. Provided that both valsartan and amlodipine are safe in children [7, 8], ‏this affords the amlodipine/valsartan extemporaneous suspension additional value for this group of patients as well. Amlodipine and valsartan combination is available in the pharmaceutical market as film-coated tablets. No liquid formulation of this combination of active ingredients is available. Therefore, crushing of the tablet is the only choice for using this drug in patients with swallowing difficulties.

There were several attempts to make valsartan extemporaneous suspension [9, 10] and amlodipine extemporaneous suspension from available commercial tablets [11], but there were no efforts done for preparing the combined (amlodipine/valsartan) suspension. However, no bioequivalence studies are conducted in such situations, which leads to further confusion whether these crushed tablets preserve their efficacy or this action may lead to serious complications.

Recently, *in silico* modeling plays an important role in the prediction of *in vivo* behavior based on *in vitro* data [12]; by the estimation of specific parameters, the computational simulation technology has proven its usefulness in pharmaceutical sciences especially in predicting the *in vivo* performance of a drug and accordingly *in vitro*–*in vivo* correlation (IVIVC) can be established [12, 13].

Therefore, the aims of this study were to develop an extemporaneous suspension of amlodipine and valsartan as a combination using crushed commercial tablets and to evaluate *in vitro* behavior of this combination in this formulation (suspension of crushed tablets) as well as to predict the *in vivo* performance of this formulation by using simulation technology based on the *in vitro* data and physicochemical properties of the drug.

## 2. Methods

### 2.1. Materials and Chemicals

Valzadepine^®^ 80/5 mg tablets (batch 036B16; expiry date 02/2018), amlodipine and valsartan USP reference standards, and all the excipients and materials (aspartame, mannitol, trisodium citrate, guar gum, potassium dihydrogen phosphate, sodium hydroxide, and glacial acetic acid) were kindly donated by Pharmacare PLC, Ramallah, Palestine.

High-performance liquid chromatography (HPLC) grade solvents of acetonitrile (ACN) and methanol (MeOH) were purchased from Carlo Erba (DASIT Group). Triethylamine and orthophosphoric acid were purchased from Merck. High purified water was prepared by using a Millipore Milli-Q Plus water purification system. All other reagents were of analytical grade.

### 2.2. Formulation of the Suspension

#### 2.2.1. Preparation of the Extemporaneous Suspension

In this study, an extemporaneous suspension containing AML 5 mg/VAL 80 mg was prepared from the commercial tablet Valzadepine® (5/80), where a total of 200 tablets of Valzadepine® (AML 5 mg/VAL 80 mg) (199.6 ± 1.3, mean ± SD) were crushed to a fine powder and mixed with the excipients. The final composition of the suspension formula is listed in Table 1.

The resulting powder was divided into 20 amber glass bottles (16 g mixture powder in each 50 mL bottle), which were ready for reconstitution to form the Valzadepine 5/80 suspension/5 mL (to be completed up to 50 mL water and to be shaken well before use).

#### 2.2.2. pH Measurements

The apparent pH of the different media was determined using Mettler Toledo MP225 pH meter; each measurement was taken in triplicate.

#### 2.2.3. Viscosity Measurements

The rheological behavior of the extemporaneous suspension was measured using Brookfield viscometer over a shear rate of 90–100 s^−1^. The viscosity measurement was performed at 25°C in duplicate, and the rheogram was obtained for the selected formula.

### 2.3. Stability Study

#### 2.3.1. Chemical Stability

The stability study was conducted by storing 10 containers containing 50 mL of the extemporaneous suspension at room temperature. Another 10 bottles containing the initial powder were kept for further analysis. The suspensions were analyzed using HPLC in duplicate in a weekly manner over a period of one month. The stability of the extemporaneous suspension was determined by calculating the percentage of the drugs remaining at the end of each week.

#### 2.3.2. Physical Stability

The formulated suspension was tested for its physical properties such as pH, viscosity, appearance, and its organoleptic properties. They were tested at the time of preparation and at the end of each week over one month at room temperature.

#### 2.3.3. Microbiological Stability

*(1) Preparation of Culture Media*. A quantity of 28 g of nutrient agar dehydrated powder was dissolved in 1 L of distilled water. The prepared suspension was heated until boiling while being mixed roughly. The solution was placed in the autoclave at 125°C for 15 minutes for sterilization. Afterwards, the solution was poured in already-sterilized Petri dishes. The Petri dishes were placed in the refrigerator for 24 hrs.

*(2) Microbiological Analysis*. After 24 hrs, 0.1 mL of each reconstituted suspension was placed on one of the Petri dishes and they were placed in the incubator at 37°C for 48 hrs. The analysis includes total bacterial count and examination of the presence of molds, yeast, *Staphylococcus aureus*, *Pseudomonas aeruginosa*, and *Candida albicans.*

### 2.4. Drug Release Study

#### 2.4.1. Dissolution

Dissolution rotating paddle apparatus II (Erweka DT70, Germany) was used to study the release of AML/VAL from the tablets as well as the extemporaneous suspension. Dissolution vessels were filled with 1000 mL each with medium and the paddle was rotating at 75 revolutions per minute (rpm) for 30 minutes; the temperature was set at (37°C ± 0.5°C).

Samples of 10 mL were withdrawn at predetermined time points, 5, 10, 15, 20, and 30 minutes, and replaced with fresh media; the samples were taken from the midway between the surface and the top of the rotating paddles not less than 1 cm from the vessel wall. Each sample was filtered through a 0.45 *µ*m microporous PTFE syringe filter; then they were injected to an HPLC instrument to quantify AML/VAL concentration in the samples [15].

### 2.5. Statistical Analysis

Similarity and difference factors (*f*_*2*_ and *f*_*1*_, respectively) were used to assess the dissolution data as reported in equations (1) and (2) below. The *f*_2_ factor is a measure of the closeness of two profiles while *f*_1_ is a measure of the difference between two profiles:

(1) $$\begin{array}{c} f_{1}=\left\{\left[\frac{\sum_{t=1}^{n}R_{t}-T_{t}}{\sum_{t=1}^{n}R_{t}}\right]\right\}\times 100, \end{array}$$

(2) $$\begin{array}{c} f_{2}=50\cdot \;\log\left\{{\left[1+\frac{1}{n}\sum_{t=1}^{n}\left(R_{t}-T_{t}\right)\right]}^{-0.5}\times 100\right\}, \end{array}$$

where *R*_*t*_ and *T*_*t*_ are the percentages of drug dissolved at each time point for the reference and test products, respectively. When *f*_1_ value is greater than 15, this indicates no similarity, and when *f*_2_ value is greater than 50, then there is a significant similarity between the two products.

### 2.6. The HPLC Analysis

#### 2.6.1. Mobile Phase Preparation

Mobile phase was prepared by mixing 2 solutions, A and B, in (1 : 1) volume ratio in which solution A is methanol, acetonitrile, and buffer (175 : 75 : 250 v/v/v), and solution B is water, acetonitrile, and glacial acetic acid (150 : 350 : 0.5 v/v/v), and the buffer was prepared by adding 7.0 mL of triethylamine into 1000 mL flask containing 900 mL of water; the pH of this buffer was adjusted to 3.0 ± 1 with phosphoric acid; then it was diluted with water to the final volume of 1000 mL. The mobile phase was filtered through a 0.45 *µ*m microporous filter and degassed by sonication prior to use.

#### 2.6.2. Instruments and Chromatographic Conditions

The HPLC system consisted of LaChrom (Merck Hitachi) equipped with model L-7100 pump, L-7200 autosampler, L-7300 column oven, DADL-7450 photodiode array (PDA) detector, and D-7000 software HSM version 3.1 (Merck Hitachi, Kent, England). The HPLC experimental conditions were optimized on a stainless steel column (250 cm × 4.6 mm) packed with octadecylsilyl (C_18_) silica gel for chromatography (5 *µ*m). The flow rate was 1.0 mL/minute with injection volume of 20 *µ*L, and the UV-detector was set to 220 nm.

#### 2.6.3. Standard Solutions Preparation

The standard solution of AML was prepared by dissolving 27.74 mg of AML besylate reference standard in diluent (ACN : Milli-Q water, 1 : 1) till reaching 200 mL; the standard solution of VAL was prepared by dissolving 80 mg of VAL reference standard in 40 mL diluents then sonicated till dissolved and the volume completed to 50 mL with the diluent. Then, the standard solution of the combination was prepared by taking 5 mL of each standard solution to 50 mL volumetric flask together and completed to 50 mL with the mobile phase.

#### 2.6.4. Working Sample Solution

Sample solution was prepared by mixing 5.5 grams of the suspension into a 50 mL volumetric flask with 10 mL of water and 30 mL of diluent, stirred, and sonicated then completed to the volume with the diluent, 5 mL of this sample solution was diluted 10 times with the mobile phase, and each sample was filtered through 0.45 *µ*m syringe tip filter. The peak area was used for quantification and comparing sample and standard peak area ratios as a function of concentration.

### 2.7. Gastrointestinal Simulation

GastroPlus™ software (version 9.0, Simulations Plus Inc., Lancaster, CA, USA), which is based on the Advanced Compartmental Absorption and Transit (ACAT), was used in this study. The approach used was to develop and verify absorption models for both AML and VAL from Valzadepine® tablet. The *in silico* models were initially constructed for immediate release (IR) tablet and were afterwards implemented to predict the *in vivo* profiles for both drugs from the extemporaneous suspension.

Therefore, two databases were established: one for AML and the other for VAL. Each database consists of two records, one for the tablet and the other for the suspension.

GastroPlus™ as a single simulation mode was used to run the gastrointestinal simulation depending on the physicochemical, physiological, and the pharmacokinetics properties of AML and VAL, as well as the *in vitro* dissolution data from both the tablet and the suspension. GastroPlus™ includes three modules: compound, physiology, and pharmacokinetics. For the compound and pharmacokinetics modules, the input data were collected from the literature. In the physiology module, the simulations were conducted using The Human Physiology Fasted mode. All the physiological parameters were fixed at default values. In the pharmacokinetic module, two compartment kinetics were followed for AML and for VAL as well; both exhibited zero-order absorption and first-order elimination [16].

The simulations were conducted using the Johnson model as a dissolution model (IR tablet) mode, and GastroPlus™ was selected for simulations. The model for IR tablet was verified by comparing the simulated profiles to the observed *in vivo* pharmacokinetic profiles of Valzadepine® tablet, which was obtained from Pharmacare Ltd. The developed model for the “IR tablet” dosage form was then employed for predicting the *in vivo* performance of the suspension. The simulation of the suspension was performed using the “IR suspension” as the selected dosage form and by introducing the dissolution data for the formulated suspension. The experimental *in vitro* dissolution profiles for both active ingredients from Valzadepine^®^ tablets and suspension in the different pH media were incorporated in the corresponding model. The summary of all input parameters for simulation is given in Table 2.

The percent of prediction error of the simulation (*%PE*) can be calculated by equation (3) below; this represents the percent of error between the predicted values and that of the *in vivo* observed data:

(3) $$\begin{array}{c} \%PE=\frac{PF_{\text{Predicted}}-PK_{\text{observed}}}{PK_{\text{observed}}}\times 100\%. \end{array}$$

## 3. Results and Discussion

For paediatric or geriatric patients with swallowing difficulties, the liquid preparations are the most convenient ones. A wide variety of medications in the pharmaceutical market are lacking the liquid oral dosage forms. Therefore, many researchers, professionals, and health care workers tend to prepare extemporaneous suspensions to cover up the shortage in the pharmaceutical market especially for paediatric medications [1].

Conroy et al. [28] reported that about 65% of medications that are used in an intensive care unit of children's hospital are off-label or unlicensed [28]. Paediatric patients are considered therapeutic orphans especially with the large decrease in medications bearing labels for paediatric administration combined with the insufficient safety data making their prescription and use limited as off-label medications [29–31]. Such medications are not registered or approved by FDA. Moreover, no bioequivalence studies are conducted in such situations, which make these suspensions questionable in terms of efficacy and safety. The combination of AML and VAL as antihypertensive medications is an example of such medications with no liquid oral formulation available.

### 3.1. Formulation and Suspension Compounding

An extemporaneous suspension of a combination of AML and VAL was successfully prepared from available commercial tablets Valzadepine® 5/80 mg as a source of the active ingredients. The AML/VAL suspension was well suspended upon brief shaking with acceptable appearance, smell, and palatable taste with a pH value of 5.5.

A sugar-free 5/80 mg AML/VAL per 5 mL suspension in a 50 mL bottle was adopted upon patient usual dose as well as stability period after reconstitution of the suspension, which is convenient for patients who have a coexisting diabetes as well. The usual daily dose of AML/VAL combination (5/80 mg) can be obtained in 5 mL of this extemporaneous suspension; the bottle (50 mL) will be sufficient for 10 days period through which the suspension is still stable and effective. Provided that the liquid preparations like this suspension provide flexible dosing capacity with the ability of administration of parts of the dose, a volume of 50 mL was chosen as a final volume of this suspension in consideration of paediatric hypertensive patients for which the amount will be saved for longer period when parts of the 5 mL dose will be given notifying them to discard the remaining amount at the fourth week after reconstitution.

### 3.2. Viscosity Determination

**Figure 1:**
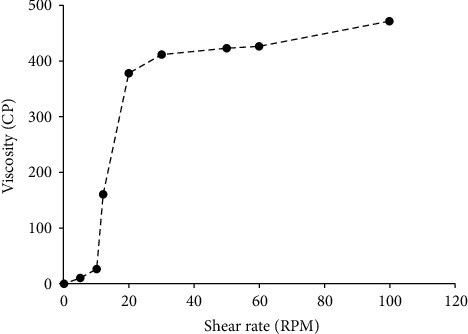
The rheological behavior of the extemporaneous preparation over different shear rates.

The viscosity of the extemporaneous suspension was examined at different shear rates. The behavior is shown to be dilatant, i.e., the viscosity increases with the increase in the shear rate. The rheological performance and viscosity data are shown in Figure 1.

### 3.3. Drug Release Study

**Figure 2:**
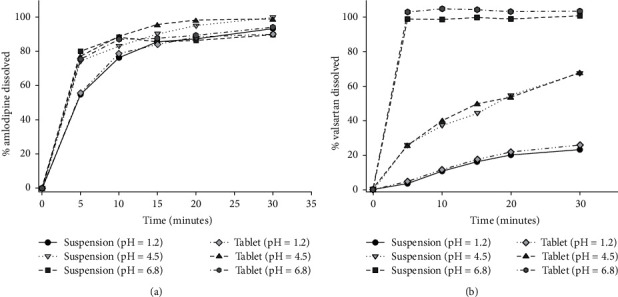
Release profiles of AML from the tablet and the suspension at different pH values (a) and of VAL from the tablet and the suspension at different pH values (b).

The *in vitro* release of AML from both the IR tablet and the suspension was investigated in media with different pH (1.2, 4.5, and 6.8). The dissolution profiles for AML from both formulations are shown in Figure 2(a). AML exhibited very rapid dissolution in phosphate buffers (4.5 and 6.8) with more than 85% being dissolved within 15 minutes, and it has a rapid dissolution in 0.1 N HCl with more than 85% being dissolved within 30 minutes and an *f*_2_ value of 51.74, which is expected for a BCS class 1 medication.Whereas for VAL, media pH has shown to have a marked effect on its release from both dosage forms (Figure 2(b)). At pH = 6.8, the percentage of VAL released was more than 85% within 15 minutes; however, at pH 4.5 and 1.2 media, dissolution was much slower. At pH of 4.5, less than 70% of the drug released within 30 minutes with *f*_*2*_ and *f*_*1*_ values being 51.63 and 3.83, respectively, whereas at pH = 1.2, the apparent amount of VAL released was not more than 26% within 30 minutes from both dosage with *f*_2_ and *f*_1_ values being 51.80 and 8.68, respectively. This decrease in the dissolution rates with the reduction in the media pH reflects the pH-dependent solubility of VAL, where the IR tablet was the reference and the extemporaneous suspension was the test. These findings are in agreement with previous studies which reported a reduction in VAL solubility at lower pH values [32–34]. The results of similarity were more than 50 for each dissolution showed latency in 85% within 15 minutes indicating the similarity in the release from both formulations.  Table 3 summarizes these findings.

According to FDA guidlines [15], concerning dissolution test for both AML and VAL tablet dosage forms, dissolution test should be carried out at a pH of 6.8. AML was found to dissolve very rapidly with an average of 87.3% and 88.1% was dissolved within 10 minutes from the tablet and the suspension, respectively.

According to BCS, AML is highly soluble and highly permeable as a BCS class 1 member; then a biowaiver is granted [35]. Unlikely, VAL BSC classification sparks a debate among researchers. Some literature considered VAL as BCS class 2 in which it must have a high permeability and low solubility due to the shortage of VAL solubility at low pH levels [36, 37]; others considered VAL as a special case with pH-dependent solubility taking into consideration that VAL solubility increases 1000 folds when pH increases from 4 to 6 [38], keeping in mind that VAL site of absorption is the upper gastrointestinal tract where it remains ionized [39] and hence barely absorbed with fraction of dose absorbed and systemically available after oral administration of about 0.23 [40]. Then, it is more likely to be BCS class 3 [41] with high solubility and low permeability [38]. To be more precise, VAL can be identified as intermediate class 2/3 rather than class 2 or class 3 as it is suggested by Chi-Yuan and Wu and Leslie Z. Benet for ciprofloxacin and erythromycin [41]. Similar situation was identified by Arthur Okumu and others for assigning etoricoxib as intermediate class 1/2 [42]. Accordingly, VAL is eligible for biowaiver if the release of VAL exceeds 85% within 15 minutes as it is suggested by BCS [43].

Nevertheless, the extent of VAL release from this extemporaneous suspension is in agreement with Zaid et al.'s study conducted on VAL extemporaneous suspension prepared from commercial tablets in which more than 85% of VAL was released within 10 minutes [10].

### 3.4. Stability Study

#### 3.4.1. Physical Stability

There were no changes observed in the appearance, taste, odour, colour, and pH.

#### 3.4.2. Chemical Stability

The suspension was chemically stable throughout the four-week period. The mean percentages of the remaining active ingredients were over 90% within the four-week period (Table 4). The mean concentrations of AML and VAL on the last day of the period were 97.3% and 101.1%, respectively, at room temperature.

#### 3.4.3. Microbial Stability

The formulated AML/VAL suspension passed the microbial testing study through the four-week period. No microbial contamination was observed in the suspension during the study period. Moreover, no changes in the appearance, pH, colour, or odour were observed; no microbial growth was detected as well throughout the 4-week period.

### 3.5. Drug Absorption Simulation

#### 3.5.1. Gastrointestinal Simulation

**Figure 3:**
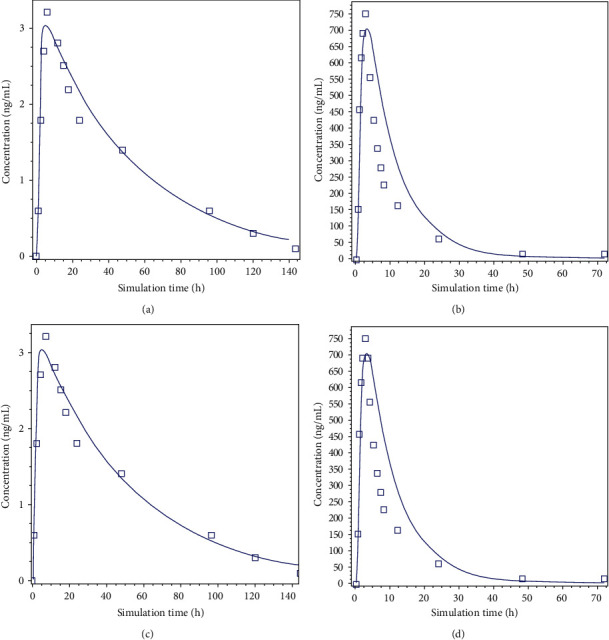
The simulated plasma profile of amlodipine 5 mg (a), valsartan 80 mg (b) obtained from Valzadepine® tablet dosage form, amlodipine extemporaneous suspension (c), and valsartan suspension (d) (__: *in silico*; □: *in vivo* data).

*In silico* simulation was used to build models describing the *in vivo* absorption of both AML and VAL from IR tablet based upon the physicochemical, physiological, and the *in vitro* dissolution data. The simulated plasma profiles for AML and VAL together with the *in vivo* observed curves following the intake of Valzadepine® IR tablet are presented in Figures 3(a) and 3(b). The simulated profiles for both drugs from the solid dosage form were in good agreement with the *in vivo* observed curves. The simulated and the *in vivo* pharmacokinetic parameters (*C*_max_ and AUC_0-∞_) for both drugs are presented in Table 5. The percent prediction errors obtained were less than 10% for all pharmacokinetic parameters, indicating good predictability. The developed models for the IR tablet dosage form were implemented to predict the *in vivo* performance of the extemporaneous suspension using the *in vitro* dissolution data of the suspension. Figures 3(c) and 3(d) for AML and VAL, respectively, compare the predicted absorption profiles for the suspension and the *in vivo* plasma profile observed for IR tablet. Then, the *in silico* pharmacokinetic parameters for suspension were compared with those observed *in vivo* for IR tablet. The extemporaneous preparation is predicted to be bioequivalent with the IR tablet dosage form, since the 90% confidence intervals for *C*_max_ and AUC_0-∞_ for both active ingredients fall within the limits of 80–125% for IR release tablet. The pharmacokinetics parameters that are predicted by the *in silico* method for suspension indicate good predictability with the percent of prediction error maintained less than 10%, the data, and predicted profiles.

*In silico* modeling provided a new insight into the prediction of bioavailability depending on *in vitro* dissolution testing [12], in which *in silico* method beside *in vitro* dissolution could be a valuable and reliable tool in predicting the bioavailability of new dosage forms and in this work for our extemporaneous compounded suspension. Arthur okumu and others suggested that similar *in vitro* dissolution profiles could justify a biowaiver when they are in compliance with *in silico* predictive profiles [42].

#### 3.5.2. Bioequivalence

Considering FDA regulations, two products are said to be bioequivalent if the 90% confidence interval (CI) of the relative mean *C*_max_ and AUC_0-∞_ of the test product to reference product is within 80–125% range [44]. In this study, the 90% CI of the geometric mean ratios (test: reference) for bioequivalent analysis obtained from pharmacokinetic parameters (*C*_max_ and AUC_0-∞_) of AML/VAL 5/80 mg extemporaneous suspension were predicted by GastroPlus^TM^ and compared to that observed for the tablet considering the suspension as the test where the tablet is the reference in order to investigate bioequivalence. The 90% CI of both pharmacokinetic parameters (*C*_max_ and AUC_0-∞_) for both drug substances lied within the acceptance criteria of FDA [80%–125%]. Depending on the simulation data and the *in vitro* dissolution data combined with bioequivalence terms that are achieved, then the compounded suspension of AML/VAL appears to be bioequivalent to the commercial tablet Valzadepine® as both are giving similar profiles that provide efficient insight into *in vivo* behavior of this extemporaneous suspension.

## 4. Conclusions

The extemporaneous suspension could be successfully prepared using available commercial tablets as a source of the active ingredients even for the combinations medications. Such suspensions should be carefully evaluated in different aspects, volume, organoleptic, stability, and bioavailability, which are lacking in such circumstances. AML/VAL extemporaneous suspension can preserve its stability over four-week period when stored at room temperature; *in silico* modeling could be applied adjacent to *in vitro* testing to predict PKs and prove similarity of an extemporaneous suspension with the tablets. Pharmaceutical companies should include a section in their leaflets regarding the compounding and stability of those suspensions when the alternative liquid dosage form is not available in the market which could be life-saving for a patient in need.

## Figures and Tables

**Table 1:** The composition of the AML/VAL 5/80 suspension formula^∗^.

Material	Function	Amount (g)
Valzadepine® crushed tab 80/5	Active ingredients	40.00
Aspartame	Sweetening agent^∗^	2.00
Mannitol	Flavoring agent^∗^	272.00
Trisodium citrate	pH modifier/buffering agent^∗^	3.20
Sodium hydroxide	pH modifier/buffering agent^∗^	0.20
Guar gum	Suspending agent^∗^	3.00
Total weight		320.40

^∗^From [14].

**Table 2:** Simulation input data.

Parameter	Value	
	Amlodipine (as besylate)	Valsartan
Molecular weight (g/mole)	567.051	435.53
Partition/distribution coefficient	2.66 (pH = 7.4)^a^	−0.34 (pH = 7)^b^
Pka_1_	8.7^c^	3.9^d^
Pka_2_	—	4.73^d^
Solubility (mg/ml)	0.774 (pH 7.4)^e^	16.8 (pH = 8)^f^
P_eff_ (human jejunal permeability) (cm/sec)	0.0743 ∗ 10^−4g^ (caco-2)	0.262 ∗ 10^−4h^ (rat)
Dose (mg)	5	80
Dose volume (ml)	250	250
Mean precipitation time (sec)	900^i^	900^i^
Diffusion coefficient (cm^2^/s)	4.2 ∗ 10^−8j^	1.1 ∗ 10^−8k^
Drug particle density (g/ml)	1.2^i^	1.2^i^
Blood plasma concentration ratio	1^i^	1^i^
Body weight (kg)	70	70
Unbound percent in plasma (%)	2^l^	5^m^
Clearance (l/hr)	28^n^	f^n^
Volume of distribution, Vc (L/Kg)	17^n^	0.23^n^
Elimination half-life (h)	27.03^o^	5.58^o^
Simulation time (hr)	144	72

^a^From [17, 18], ^b^from [19], ^c^from [20, 21], ^d,f^from[22], ^e^from [23], ^g^ from[18], ^h^from [24], ^i^from GastroPlus default values, ^j, k^from[25], ^l^from [26], ^m^from [27], ^n^GastroPlus calculated using PBPKPlus™ module, and ^o^GastroPlus calculated (built-in calculation from PK parameters).

**Table 3:** Dissolution of AML and VAL from Valzadepine® tablets and extemporaneous suspension at different pH values and two time points.

	Tablet		Suspension	
Medium	15 min	30 min	15 min	30 min
% Dissolved of amlodipine ± (SD)				
pH 1.2	84.4 ± (2.14)	90.3 ± (1.81) (*f*_2_ = 51.74)	85.6 ± (1.32)	93.3 ± (1.59)
pH 4.5	95.6 ± (1.67)	98.7 ± (1.73)	90.4 ± (1.68)	100.1 ± (1.97)
pH 6.8	87.8 ± (1.97)	94.3 ± (0.78)	87.1 ± (0.87)	89.9 ± (1.05)

% Dissolved of valsartan ± (SD)				
pH 1.2	17.7 ± (2.07)	25.8 ± (1.91) (*f*_2_ = 51.80)	16.1 ± (1.96)	23.5 ± (1.45)
pH 4.5	49.6 ± (2.18)	68.1 ± (1.89) (*f*_2_ = 51.63)	44.4 ± (1.84)	67.9 ± (2.76)
pH 6.8	104.4 ± (1.83)	103.4 ± (1.22)	99.8 ± (0.56)	100.7 ± (1.74)

**Table 4:** The mean percentage of the active ingredient in AML/VAL suspension throughout 4-week period at room temperature.

Weak	% of remaining drug	
	AML	VAL
Initial	102.1	106.2
Week 1	101.8	105.3
Week 2	99.1	102.2
Week 3	98.3	101.9
Week 4	97.3	101.1

**Table 5:** Observed and predicted pharmacokinetic parameters with percentage of prediction error for both amlodipine and valsartan.

		Pharmacokinetic parameter	
		*C*_max_ (ng/mL)	AUC_0-∞_ (ng.hr/mL)
Amlodipine	Calculated for tablet	3.030, PE = 5.306%	169.81, PE = 5.76%
	Observed	3.200	160.65
	Calculated for suspension	3.027, PE = 5.406%	169.78, PE = 5.74%

Valsartan	Calculated for tablet	704.55, PE = 5.72%	8517.70, PE = 4,826%
	Observed	747.30	8949.70
	Calculated for suspension	707.22, PE = 5,36%	8517.60, PE = 4,82%

## Data Availability

All raw data are available upon request from the corresponding author.

## Additional Points

*Summary*. Amlodipine/valsartan extemporaneous suspension was prepared from available commercial tablets with acceptable organoleptic properties. The dissolution profiles for the extemporaneous preparation and the commercial tablets were determined in different pH media. The physical, chemical, and microbial stability of the compounded formulation was evaluated over one-month period at room temperature. The suspension was stable over the studied period. *In silico* modeling using GastroPlus^TM^ software was conducted. The release profiles of valsartan and amlodipine from the suspension were similar to those from source tablet. *In silico* modeling predicted similarity of the extemporaneous suspension and the commercial tablets.

## References

[1] Abdallah Y. M. M. (2015). *Attitudes, Knowledge and Practices of Health-Care Practitioners toward Splitting or Crushing Oral Solid Dosage Forms in Palestine: Safety and Therapeutic Implications*.

[2] Tahaineh L. M., Gharaibeh S. F. (2012). Tablet splitting and weight uniformity of half-tablets of 4 medications in pharmacy practice. *Journal of Pharmacy Practice*.

[3] Glass B. D., Haywood A. (2006). Stability considerations in liquid dosage forms extemporaneously prepared from commercially available products. *Journal of Pharmacy and Pharmaceutical Sciences*.

[4] Burnier M., Brunner H. (2000). Angiotensin II receptor antagonists. *The Lancet*.

[5] Flack J. M., Calhoun D. A., Satlin L., Barbier M., Hilkert R., Brunel P. (2009). Efficacy and safety of initial combination therapy with amlodipine/valsartan compared with amlodipine monotherapy in black patients with stage 2 hypertension: the EX-STAND study. *Journal of Human Hypertension*.

[6] Philipp T., Smith T. R., Glazer R. (2007). Two multicenter, 8-week, randomized, double-blind, placebo-controlled, parallel-group studies evaluating the efficacy and tolerability of amlodipine and valsartan in combination and as monotherapy in adult patients with mild to moderate essential hypertension. *Clinical Therapeutics*.

[7] Flynn J. T., Smoyer W. E., Bunchman T. E. (2000). Treatment of hypertensive children with amlodipine. *American Journal of Hypertension*.

[8] Wells T., Blumer J., Meyers K. E. C. (2011). Effectiveness and safety of valsartan in children aged 6 to 16 years with hypertension. *The Journal of Clinical Hypertension*.

[9] Talamonti W., Wagner R. F., Wen H. (2008). *Pharmaceutical Formulation of Valsartan*.

[10] Zaid A. N., Assali M., Qaddomi A., Ghanem M., Zaaror Y. A. (2013). Preparation and stability evaluation of extemporaneous oral suspension of valsartan using commercially available tablets. *International Journal of Pharmaceutical Compounding*.

[11] Lyszkiewicz D. A., Levichek Z., Kozer E. (2003). Bioavailability of a pediatric amlodipine suspension. *Pediatric Nephrology*.

[12] Honório T. d. S., Pinto E. C., Rocha H. V. A. (2013). In vitro-in vivo correlation of efavirenz tablets using GastroPlus. *AAPS PharmSciTech*.

[13] Van De Waterbeemd H., Gifford E. (2003). ADMET in silico modelling: towards prediction paradise?. *Nature Reviews Drug Discovery*.

[14] Kulshreshtha A. K., Singh O. N., Wall G. M. (2010). *Pharmaceutical Suspensions*.

[15] Pharmacopeia U. S., national formulary U.-N. (2017). *United States Pharmacopeia and National Formulary (USP 40- NF 35)*.

[16] Heo Y.-A., Holford N., Kim Y., Son M., Park K. (2016). Quantitative model for the blood pressure-lowering interaction of valsartan and amlodipine. *British Journal of Clinical Pharmacology*.

[17] Caron G., Ermondi G., Damiano A. (2004). Ionization, lipophilicity, and molecular modeling to investigate permeability and other biological properties of amlodipine. *Bioorganic and Medicinal Chemistry*.

[18] Patel H. J., Patel J. S., Desai B., Patel K. D. (2010). Permeability studies of anti hypertensive drug amlodipine besilate for transdermal delivery. *Asian Journal of Pharmaceutical and Clinical Research*.

[19] Yamashiro W., Maeda K., Hirouchi M., Adachi Y., Hu Z., Sugiyama Y. (2006). Involvement of transporters in the hepatic uptake and biliary excretion of valsartan, a selective antagonist of the angiotensin II AT1-receptor, in humans. *Drug Metabolism and Disposition*.

[20] Burges R., Moisey D. (1994). Unique pharmacologic properties of amlodipine. *The American Journal of Cardiology*.

[21] Van Zwieten P. (1994). Amlodipine: an overview of its pharmacodynamic and pharmacokinetic properties. *Clinical Cardiology*.

[22] Criscione L., Bradley W. A., Bühlmayer P. (1995). Valsartan: preclinical and clinical profile of an antihypertensive angiotensin-II antagonist. *Cardiovascular Drug Reviews*.

[23] McDaid D. M., Deasy P. B. (1996). Formulation development of a transdermal drug delivery system for amlodipine base. *International Journal of Pharmaceutics*.

[24] Lozoya‐Agullo I., González‐Álvarez I., González‐Álvarez M., Merino‐sanjuán M., Bermejo M. (2015). In situ perfusion model in rat colon for drug absorption studies: comparison with small intestine and caco‐2 cell model. *Journal of Pharmaceutical Sciences*.

[25] Erden P. E., Taşdemir İH., Kaçar C., Kılıç E. (2014). Simultaneous determination of valsartan and amlodipine besylate in human serum and pharmaceutical dosage forms by voltammetry. *International Journal of Electrochemical Science*.

[26] Meredith P. A., Elliott H. L. (1992). Clinical pharmacokinetics of amlodipine. *Clinical Pharmacokinetics*.

[27] Thürmann P. A. (2000). Valsartan: a novel angiotensin type 1 receptor antagonist. *Expert Opinion on Pharmacotherapy*.

[28] Conroy S., McIntyre J., Choonara I., Hull P. S. D. (1999). Unlicensed and off label drug use in neonates Commentary. *Archives of Disease in Childhood-Fetal and Neonatal Edition*.

[29] Ali A. A., Charoo N. A., Abdallah D. B. (2014). Pediatric drug development: formulation considerations. *Drug Development and Industrial Pharmacy*.

[30] Nahata M. C. (1999). Lack of pediatric drug formulations. *Pediatrics*.

[31] Rakhmanina N., Vandenanker J. (2006). Pharmacological research in pediatrics: from neonates to adolescents☆. *Advanced Drug Delivery Reviews*.

[32] Chella N., Shastri N., Tadikonda R. R. (2012). Use of the liquisolid compact technique for improvement of the dissolution rate of valsartan. *Acta Pharmaceutica Sinica B*.

[33] Dixit A. R., Rajput S. J., Patel S. G. (2010). Preparation and bioavailability assessment of SMEDDS containing valsartan. *AAPS Pharmscitech*.

[34] Biswas N., Kuotsu K. (2017). Chronotherapeutically modulated pulsatile system of valsartan nanocrystals-an in vitro and in vivo evaluation. *AAPS PharmSciTech*.

[35] Food Administration (2000). *Guidance for Industry: Waiver of in Vivo Bioavailability and Bioequivalence Studies for Immediate-Release Solid Oral Dosage Forms Based on a Biopharmaceutics Classification System*.

[36] Cappello B., Maio C. D., Iervolino M., Miro A. (2006). Improvement of solubility and stability of valsartan by hydroxypropyl-\boldbeta-cyclodextrin. *Journal of Inclusion Phenomena and Macrocyclic Chemistry*.

[37] Pradhan R., Kim S. Y., Yong C. S., Kim J. O. (2016). Preparation and characterization of spray-dried valsartan-loaded Eudragit E PO solid dispersion microparticles. *Asian Journal of Pharmaceutical Sciences*.

[38] Saydam M., Takka S. (2007). Bioavailability file: valsartan. *FABAD Journal of Pharmaceutical Sciences*.

[39] Siddiqui N., Husain A., Chaudhry L., Alam M. S., Mitra M., Bhasin P. S. (2011). Pharmacological and pharmaceutical profile of valsartan: a review. * Journal of Applied Pharmaceutical Science*.

[40] Flesch G., Müller P., Lloyd P. (1997). Absolute bioavailability and pharmacokinetics of valsartan, an angiotensin II receptor antagonist, in man. *European Journal of Clinical Pharmacology*.

[41] Wu C.-Y., Benet L. Z. (2005). Predicting drug disposition via application of BCS: transport/absorption/elimination interplay and development of a biopharmaceutics drug disposition classification system. *Pharmaceutical Research*.

[42] Okumu A., DiMaso M., Löbenberg R. (2009). Computer simulations using GastroPlus to justify a biowaiver for etoricoxib solid oral drug products. *European Journal of Pharmaceutics and Biopharmaceutics*.

[43] Cheng C.-L., Yu L. X., Lee H.-L., Yang C.-Y., Lue C.-S., Chou C.-H. (2004). Biowaiver extension potential to BCS Class III high solubility-low permeability drugs: bridging evidence for metformin immediate-release tablet. *European Journal of Pharmaceutical Sciences*.

[44] Food Administration (2003). *Guidance for Industry: Bioavailability and Bioequivalence Studies for Orally Administered Drug Products—General Considerations*.

